# The MAP kinase negative regulator DUSP2 (dual specificity phosphatase 2) is controlled by oncogenic microRNA cluster miR-17-92, miR-106a-363 and miR-106b-25

**DOI:** 10.1186/s12885-025-14434-z

**Published:** 2025-06-19

**Authors:** Victoria Tenhaken, Ole-Morten Seternes, Ingolf Cascorbi, Henrike Bruckmueller

**Affiliations:** 1 Institute of Experimental and Clinical Pharmacology, University Hospital Schleswig-Holstein, Campus Kiel, Arnold-Heller-Str. 3, 24105 Kiel, Germany; 2 Department of Pharmacy, UiT The Arctic University of Norway, Tromsø, 9037 Norway

**Keywords:** Phosphatase, MicroRNA, Cancer, DUSP2, MAPK

## Abstract

**Background:**

Aberrant changes in protein phosphorylation are a hallmark of cancer, often leading to hyperactivation of signalling pathways such as the mitogen activated protein kinase (MAPK) pathway. Although kinase inhibitors are successfully used in certain clinical indications, drug resistance remains a challenge, and alternative approaches to control phosphorylation-dependent oncogenic signalling are increasingly being considered. These include the modulation of negative regulators of oncogenic signalling pathways. The dual-specificity phosphatase 2 (DUSP2) is one of the essential negative regulators for the MAPK pathway, providing tight and efficient control of MAPKs under physiological conditions. However, in oncogenic contexts, negative feedback regulation is often impaired and the mechanisms controlling DUSP2 expression and function remain largely elusive. The aim of the present study was to investigate whether microRNA-mediated regulation of DUSP2 could contribute to an impairment of negative feedback regulation in cancer.

**Methods:**

A combination of in silico target prediction, integrative analysis of pan-cancer microRNA and *DUSP2* mRNA expression data as well as a literature search was applied to identify microRNAs potentially regulating *DUSP2* expression in cancer context. Predicted interactions of microRNAs with the *DUSP2* 3’UTR were verified using reporter gene assays and functionally validated in a lymphoma cell model.

**Results:**

A comprehensive analysis of microRNA and *DUSP2* mRNA expression data across 32 cancer types revealed significant inverse correlations between oncogenic microRNA clusters (miR-17-92, miR-106a-363, and miR-106b-25 cluster) and *DUSP2* expression in various cancer types. Reporter gene assay analysis confirmed the interaction of miR-17-5p, miR-20a-5p, miR-20b-5p, miR-29b-3p, miR-93-5p, miR-106b-5p, miR-122-5p, miR-340-5p, miR-520a-3p, and miR-520c-3p with the *DUSP2* mRNA 3’UTR. Furthermore, treatment of the lymphoma cell line WSU-DLCL2 with microRNA inhibitors for miR-17-5p, miR-20b-5p, or miR-106b-5p resulted in increased *DUSP2* mRNA levels.

**Conclusion:**

The results of this study indicate that microRNA-mediated regulation of DUSP2 in hematologic and solid cancers appears to be a plausible mechanism that contributes to the dysregulation of MAP kinase signaling pathways in cancer by impairing negative feedback regulation. The data provide a solid foundation for future studies to investigate the consequences of regulation of DUSP function in cancer in more depth.

**Supplementary Information:**

The online version contains supplementary material available at 10.1186/s12885-025-14434-z.

## Introduction

Complex changes in several signalling pathways are hallmarks of cancer (https://gco.iarc.fr/today). Among these, the hyperactivated or mutated mitogen-activated protein kinase (MAPK) signalling pathway contributes to malignant transformation of more than 85% of human cancer types [1]. MAPKs are involved in the control of physiological processes such as proliferation, differentiation, apoptosis, and stress response as well as in cancer pathophysiology and response to cancer therapy [2]. Three main families of MAPKs are found in mammalian cells: the extracellular signal-regulated protein kinases (ERK1/2), the p38 MAP kinases (isoforms α, β, γ, δ), and the c-Jun NH2-terminal kinases (JNK1/2/3). All of them are organized into three-tier signalling cascades leading to a series of phosphorylation events that result in cellular response [2].

Since most oncogenic processes are controlled by the phosphorylation state of proteins, a revolution in cancer therapies was expected when kinase inhibitors entered the clinics. However, few kinase inhibitors provide long-term clinical benefits, while most induce rapid tolerance to treatment and rewiring of intracellular signalling pathways [2, 3]. Limited binding affinity to target proteins can lead to ineffectiveness, possibly due to mutated or constitutively phosphorylated target proteins [3]. In addition, disruption of negative feedback mechanism that attenuate proliferation can result in the activation of downstream signalling pathways, as well as evasion of drug targets through acquired oncogenic alterations [4, 5] Therefore, recent discussions have focused on alternative approaches to modify phosphorylation-dependent oncogenic signalling, including the modulation of tumour suppressor phosphatases that act as counterparts to oncogenic kinases [3].

The family of dual-specificity (Thr/Tyr) protein phosphatases (DUSPs), also termed MAP kinase phosphatases (MKPs) are major negative regulators of MAP kinase activity [6]. DUSP2 is a member of the inducible MKP subgroup that has been linked to tumour suppressor phosphatase activity. This link is supported by studies reporting low expression levels of DUSP2 in a range of cancers, including acute leukaemia, breast, colon, lung, ovary, kidney, prostate and cervical cancer [7–15]. These observations suggest that the downregulation of DUSP2 may contribute to dysregulation of key signaling pathways involved in tumorigenesis.

Furthermore, decreased DUSP2 expression is associated with poor clinical outcome and metastases formation in bladder, colorectal and serious ovarian cancer. However, it is difficult to evaluate the possible involvement of DUSP2 in tumorigenesis and treatment response due to the incomplete understanding of the molecular mechanisms controlling its expression in cancer. Thus, the recent discovery of DUSP2 as a target of post-transcriptional regulation via microRNAs is of particular of interest. MicroRNAs are short non-coding RNAs known to regulate their target genes at the post-transcriptional level by mRNA degradation or translational repression and have been linked to cancer hallmarks e.g. by downregulating tumour suppressors [4, 16–25]. Although dysregulated microRNAs have been associated with MAPK pathway activation and carcinogenesis, little is known about the role of microRNA-mediated regulation of negative MAPK pathway regulators such as DUSP2 in cancer context [26–28].

Therefore, the aim of the current study was to determine whether *DUSP2* mRNA is a target of cancer-associated microRNAs thereby contributing to the dysregulation of DUSP2 which may occur in specific cancer types. By applying a combination of in silico target prediction, integrative analysis of pan-cancer *DUSP2* mRNA expression with microRNA and MAPK phosphorylation data, and literature search microRNAs were identified and validated in vitro for their interaction with the 3’UTR of DUSP2 in a DLBCL cell line model.

## Methods

### In silico target prediction

To identify microRNAs that could potentially bind to the *DUSP2* 3’UTR, in silico target prediction was performed using the prediction tools TargetScan (Release 7.2, March 2018), Diana microT-CDS (Ensembl Version 77 miRbase v18, 07/2012), miRDB (Version 6.0, Release: June 2019) and miRTaBase (Version 7.0, Release 15.09.2017) [29–32]. MicroRNAs whose interaction was predicted by at least two prediction tools, including TargetScan, were considered for further analysis. Sequence similarity of the putative microRNA binding sites in the *DUSP2* 3´UTR was ensured by comparing the predicted binding sequences of the different prediction tools.

### Correlation analysis of *DUSP2* with microRNA expression and MAPK phosphorylation levels in the Cancer Genome Atlas Project (TCGA) samples

The interactive CancerMIRNome database (accessed 12 July 2022) was used to access and extract normalized expression data of predicted microRNAs and *DUSP2* (log_2_ transformed counts per million (CPM)) from 32 cancer types and available controls that are part of *The Cancer Genome Atlas Program* (TCGA) (www.cancer.gov/tcga) [33]. The following cancer types were included in the present study: ACC, BLCA, BRCA, CESC, CHOL, COAD, DLBC, ESCA, HNSC, KICH, KIRC, KIRP, LAML, LGG, LIHC, LUAD, LUSC, MESO, OV, PAAD, PCPG, PRAD, READ, SARC, SKCM, STAD, TGCT, THCA, THYM, UCEC, UCS, UVM (Table 1). MicroRNAs with median expression levels below 1 CPM per cancer type were excluded from further analysis. Spearman rank correlation analysis was performed on the remaining microRNAs to assess potential microRNA − *DUSP2* interactions across all 32 cancer types (GraphPad Software, Version 9.2.0, San Diego, California, USA). Only microRNA − *DUSP2* pairs showing a significant correlation (*p* < 0.05) were considered for further analysis. Differences in expression of microRNA-DUSP2 pairs between tumour samples and controls were calculated based on log_2_ transformed CPM values and expressed as log_2_FC (log_2_ fold change). Cancer types with an insufficient number of controls or no correlation of microRNAs and DUSP2 were excluded from this analysis. *DUSP2* mRNA expression levels in DLBCL and adjacent controls were also extracted using the Genevestigator database from a study by Dybkær et al. [34, 35] Spearman rank correlation analysis was also performed for *DUSP2* expression and abundance of phosphorylated MAPK proteins were determined in subsets of TCGA samples (Table 1) by Reverse Phase Protein Arrays (RPPA) and assessed via cBioPortal [36, 37].

**Table 1:** Description of TCGA cancer types analysed in the study including the number of tumour samples and controls

Cancer type abbreviation	Cancer type	Tumour samples (expression data) *n*	Tumour samples (RPPA data) *n*	Controls *n*
ACC	Adrenocortical carcinoma	79	45	–
BLCA	Bladder Urothelial Carcinoma	405	343	19
BRCA	Breast invasive carcinoma	1072	876	104
CESC	Cervical squamous cell carcinoma and endocervical adenocarcinoma	304	166	3
CHOL	Cholangiocarcinoma	36	30	9
COAD	Colon adenocarcinoma	441	346	8
DLBC	Lymphoid Neoplasm Diffuse Large B-cell Lymphoma	47	33	–
ESCA	Esophageal carcinoma	161	125	11
HNSC	Head and Neck squamous cell carcinoma	495	211	44
KICH	Kidney Chromophobe	65	62	24
KIRC	Kidney renal clear cell carcinoma	512	455	71
KIRP	Kidney renal papillary cell carcinoma	288	209	32
LAML	Acute Myeloid Leukaemia	151	-	–
LGG	Brain Lower Grade Glioma	507	428	–
LIHC	Liver hepatocellular carcinoma	367	179	50
LUAD	Lung adenocarcinoma	507	360	20
LUSC	Lung squamous cell carcinoma	475	317	38
MESO	Mesothelioma	86	63	–
OV	Ovarian serous cystadenocarcinoma	371	121	–
PAAD	Pancreatic adenocarcinoma	177	122	4
PCPG	Pheochromocytoma, Paraganglioma	178	79	3
PRAD	Prostate adenocarcinoma	491	350	52
READ	Rectum adenocarcinoma	160	26	3
SARC	Sarcoma	257	218	–
SKCM	Skin Cutaneous Melanoma	97	329	–
STAD	Stomach adenocarcinoma	372	354	32
TGCT	Testicular Germ Cell Tumours	150	118	–
THYM	Thymoma	119	90	–
THCA	Thyroid carcinoma	501	369	58
UCS	Uterine Carcinosarcoma	56	48	–
UCEC	Uterine Corpus Endometrial Carcinoma	534	423	33
UVM	Uveal Melanoma	80	12	–

* RPPA = Protein abundance data derived from reverse phase protein arrays

### Literature search

To further elucidate the role of microRNAs that showed a significantly negative correlation with *DUSP2* expression in this study, a literature search was performed in PubMed (https://pubmed.ncbi.nlm.nih.gov). The literature was searched for microRNA involvement in cancer and MAPK hyperactivation, as this could indicate a loss of negative feedback mechanisms. Based on in silico target prediction, pan-cancer correlation analysis and literature search, the following microRNAs were selected for functional validation: miR-17-5p, miR-20a-5p, miR-20b-5p, miR-29b-3p, miR-29c-3p, miR-93-5p, miR-106b-5p, miR-122-5p, miR-142-5p, miR-340-5p, and miR-373-3p, miR-520a-3p and miR-520c-3p.

### Cloning of luciferase vectors for reporter gene assays

For in vitro validation of predicted interactions between microRNAs and *DUSP2* 3’UTR, luciferase reporter gene assays were performed. The full human *DUSP2* 3’UTR (NM_004418) was cloned into the pEZX-MT01 dual luciferase expressing vector (GeneCopoeia, Rockville, Maryland, USA) using the Expand High Fidelity PCR System (Sigma-Aldrich, St. Louis, Missouri, USA). The PCR was performed with the forward primer ctagagtcggggcgcccgcgGGTGGTGCCCCTCTGCCT and the reverse primer catgtctgctcgaactagtcTTGTTGTTTTTTAAATATAACAATATTTTATT according to the manufacturer’s recommendations with 69 °C as annealing temperature. The pEZX-MT01 empty vector was digested with AsiSI and XhoI (New England Biolabs, Ipswich, Massachusetts, USA) in Cut Smart buffer according to the manufacturer’s protocol for 3 h. The assembly reaction of the DpnI digestion PCR product and the digested vector was performed according to supplier’s protocol using the NEBuilder High-Fidelity DNA Assembly Cloning Kit (New England Biolabs).

### Cell culture and transfection

HepG2 cells (ACC180, DSMZ, Braunschweig, Germany) and WSU-DLCL2 cells (ACC575, DMSZ) were cultured in RPMI1640 growth medium (Sigma-Aldrich) supplemented with 10% heat-inactivated FCS (FBC Superior Stabil, Bio&Sell, Feucht, Germany) at 37 °C and 5% CO2.

For reporter gene assay experiments HepG2 cells were transfected with precursor microRNA or control. 20 µl transfection mixture was prepared according to the manufacturer’s protocol consisting of OptiMEM (Gibco, Thermo Fisher Scientific), siPort NeoFX transfection reagent (Thermo Fisher Scientific), 10 nM of the respective pre-microRNA and vector construct (70 ng/well). 80 µl cell suspension (1250 cells/µl) was added to each well of a 96-well plate and growth medium was replaced 24 h after transfection. To determine the intracellular effect of microRNAs on *DUSP2* mRNA expression 2 × 10^6^ WSU-DLCL2 cells were transfected witheither 100nM mirVana miRNA inhibitor negative control #1 (4464076, Thermo Fisher Scientific) or the mirVana miRNA inhibitors for miR-17-5p (MH12412, Thermo Fisher Scientific), miR-20b-5p (MH10975, Thermo Fisher Scientific) or miR-106b-5p (MH10067, Thermo Fisher Scientific) using the Amaxa SE Cell Line 4D-Nucleofector X Kit S (VAXC-1032, Lonza Bioscience, Cologne, Germany) on an Amaxa 4D Nucleofector device (Lonza Bioscience) according to the manufacturer’s instructions with the CL-120 program. The cells were harvested 24 h after transfection and stored at -80 °C until subsequent RNA isolation.

### Luciferase reporter gene assays

Precursor microRNAs (pre-miRNAs) used in reporter gene assays were purchased from Thermo Fisher Scientific (Waltham, Massachusetts, USA): pre-miR-17-5p (PM12412), pre-miR-20a-5p (PM10057), pre-miR-20b-5p (PM10975), pre-miR-29b-3p (PM10103), pre-miR-29c-3p (PM10518), pre-miR-93-5p (PM10951), pre-miR-106b-5p (PM10067), pre-miR-122-5p (PM11012), pre-miR-142-5p (PM10979), pre-miR-340-5p (PM12670), pre-miR-373-3p (PM11024), pre-miR-520a-3p (PM 10391), pre-miR-520c-3p (PM12719) and pre-miRNA Precursor Negative Control #1(AM17110). Reporter gene activities were measured 48 h after transfection using the dual luciferase reporter assay system from Promega (Mannheim, Germany) on a Veritas microplate luminometer (Tuner Biosystems, Sunnyvale, CA, USA). Data analysis was carried out as described previously [38].

### Site-directed mutagenesis

To confirm the observed effects on reporter gene activity as a consequence of specific microRNAs binding to the predicted binding sites in the *DUSP2* 3’UTR, site-directed mutagenesis of the predicted binding region was performed. A restoration of the reporter gene signal in the mutated-vector compared to the wild-type vector confirmed the exact microRNA binding site. For this purpose, five bases in each of the three predicted binding sites were mutated individually. The site-directed mutated vectors were obtained from GenScript (New Jersey, USA) harbouring the following mutations in the *DUSP2* 3´UTR: MUT1: GGUGC → AUCUA, mutation site 179–183 bp; MUT2: CACTC→ AGTCA, 539–543 bp and MUT3: CUUUA→ AGCGU, mutation site 593–597 bp.

### Gene expression analysis

Total RNA was isolated using E.Z.N.A. Total RNA Kit 1 (Omega, Bio-Tek, Norcross, USA) according to the manufacturer’s recommendations and eluted in 40 µl nuclease-free water and stored at -80 °C prior to further use. Four hundred ng total RNA was reverse-transcribed using the High-Capacity cDNA Reverse Transcription Kit (Thermo Fisher Scientific) according to the manufacturer’s recommendations. The expression levels of DUSP2 (Hs00358879_m1) were determined on a QuantStudio™ 7 Flex Real-Time PCR Instrument (Thermo Fisher Scientific) using the relative gene expression protocol with TaqMan Universal Master Mix II, with UNG (#4440039, Thermo Fisher Scientific). Gene expression levels were analysed using the ΔΔCt method with GAPDH (Hs02758991_g1) and TBP (Hs00427620_m1) as endogenous controls [39].

### Statistics

Differences in microRNA and gene expression, as well as in normalized reporter gene activity were calculated using Mann-Whitney U-tests (GraphPad Software). *P*-values < 0.05 were considered as statistically significant.

## Results

### Identification of potential microRNA-*DUSP2* interactions

**Fig. 1:**
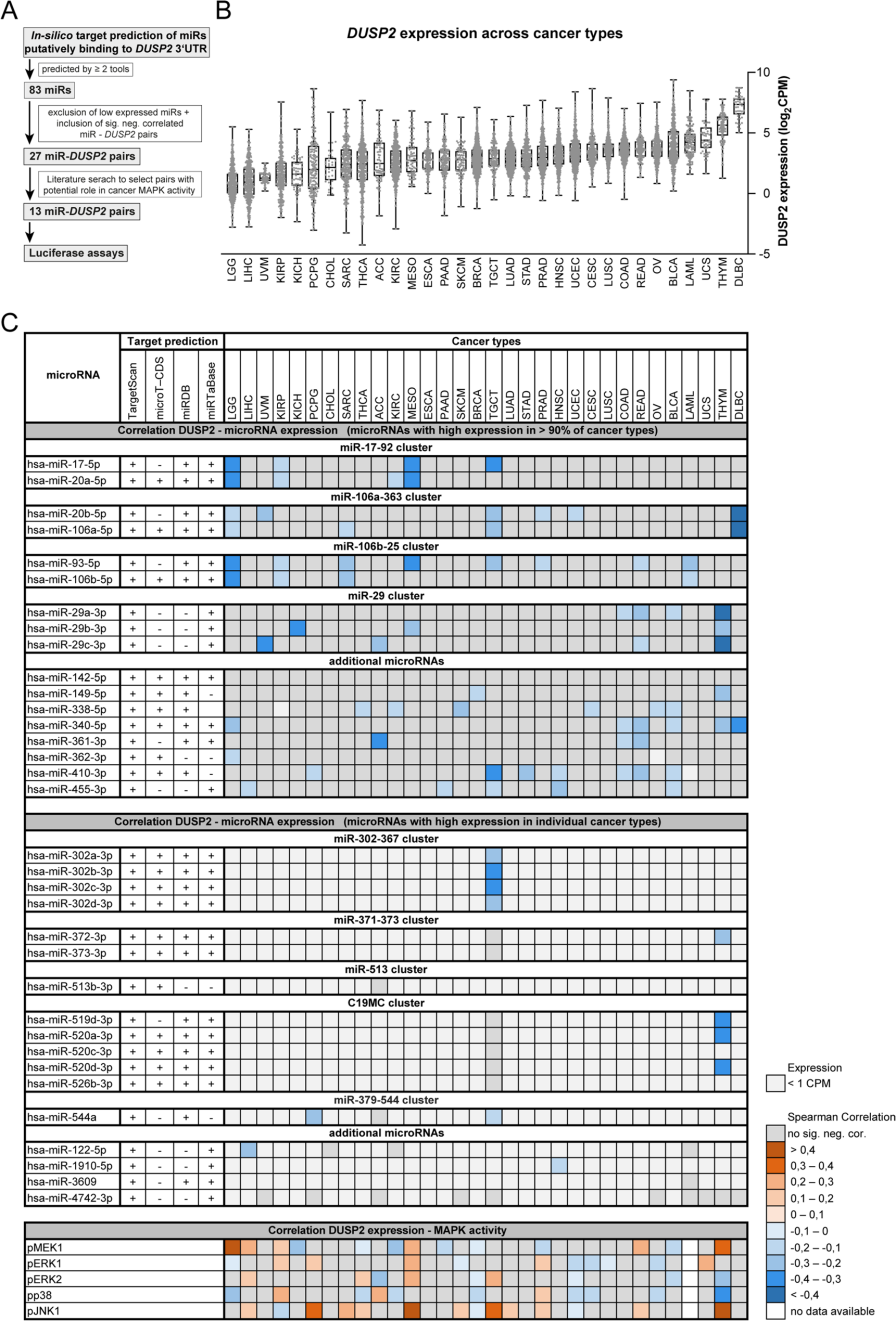
Combined approach of in silico target prediction and pan-cancer correlation analysis exhibiting all significant negatively correlated microRNAs–*DUSP2* pairs in cancer context. (**A**) Flow chart depicting the combined analysis approach for rigorous preselection of potential microRNA– *DUSP2* interactions. (**B**) Box plots showing the *DUSP2* mRNA expression levels across 32 TCGA cancer types ordered by median expression of cancer type. (**C**) In the upper part, the left column presents on top, the 17 highly expressed microRNAs (CPM > 1 in > 90% of all TCGA tumour samples) and, below, the 17 microRNAs highly expressed in individual cancer types which were predicted to bind DUSP2 at least by two prediction tools including TargetScan (middle column). The right columns present the negative Spearman correlations calculated between microRNA and *DUSP2* mRNA expression in TCGA cancer samples of respective cancer types. The lower part exhibited the Spearman correlations between *DUSP2* mRNA expression and abundance of phosphorylated MAPK proteins determined by RPPA in subsets of TCGA samples of respective cancer types. The blue colour indicates significant negative correlations, red indicates significant positive correlations, grey indicates no significant negative correlation and light grey indicated low expression < 1 CPM

To identify microRNAs that could interact with the *DUSP2* 3’UTR, a combined approach of in-silico target prediction, pan-cancer correlation analysis and literature search was conducted (Fig. 1A). First, four different in silico target prediction tools were applied identifying 402 microRNAs by TargetScan, 45 by Diana micro-T-CDS, 49 by miRDB and 47 by miRTaBase [29–32]. A total of 83 microRNAs were predicted at least by two tools, including TargetScan (Tab. S1). About one-third of these microRNAs were assigned to known microRNA clusters including the oncogenic miR-17-92 cluster (miR-17-5p, miR-20a-5p) with its paralogues the miR-106a-363 (miR-20b-5p, miR-106a-5p) and the miR-106b-25 cluster (miR-93-5p, miR-106b-5p), the miR-29 cluster (miR-29a-3p, miR-29b-3p, miR-29c-3p), the miR-302-367 cluster (miR-302a-3p, miR-302b-3p, miR-302c-3p.1, miR-302d-3p, miR-302e), the miR-371-373 cluster (miR-372-3p, miR-373-3p) and the C19MC cluster (miR-519d-3p, miR-520a-3p, miR-520b-3p, miR-520c-3p, miR-520d-3p, miR-520e, miR-526b-3p, Tab. S1).

### Evidence for microRNA-mediated DUSP2 regulation in various cancer types

The next step aimed to identify those of the 83 microRNAs being most likely involved in DUSP2 regulation in cancer context. For this purpose, microRNA and *DUSP2* expression levels were examined in silico in approximately 9,000 tumour samples of 32 cancer types from the Cancer Genome Atlas (TCGA) program (Fig. 1B, C). Seventeen microRNAs showed high expression levels in 90% of all tumour samples (CPM > 1) and most (16 microRNAs) were significantly negatively correlated to *DUSP2* mRNA expression in respective cancer types which could indicate a microRNA-induced mRNA degradation (*p* < 0.05, Fig. 1C, Tab S1) [40]. Additionally, 11 of the 17 microRNAs highly expressed in certain cancer types (CPM > 1) also exhibited significant negative correlations with *DUSP2* mRNA levels (*p* < 0.05, Fig. 1C, Tab S1). The majority of negative correlations were found for members of the oncogenic miR-17-92 cluster and its paralogues miR-106a-363 and miR-106b-25. Notable examples include lymphoma (DLBC, miR-20b-5p, *r* = -0.40, *p* = 0.005, miR-106a-5p, *r* = -0.48, *p* < 0.001) and leukaemia (LAML, miR-93-5p, *r* = -0.20, *p* < 0.01; miR-106b-5p, *r* = -0.18, *p* = 0.023) as well as solid tumours like low grade glioma (LGG, e.g. miR-17-5p, *r* = -0,34, *p* < 0.001, miR-20a-5p, *r* = 0-0.315, *p* < 0.001) or kidney cancer (KIRC, miR-20a-3p, *r* = -0.111, *p* = 0.012; KIRP, e.g. miR-93-5p, *r* = -0.196, *p* < 0.001) (Fig. 1C, Tab. S1) [41]. Expression of miR-29 cluster members correlated significantly negative to *DUSP2* mRNA levels i.a. in thymoma, while members of the C19MC cluster showed exclusively in thymoma significant negative correlations. Moreover, significant negative correlations of miR-302-367 cluster members to *DUSP2* mRNA levels were exclusively found in testicular germ cell tumours (TGCT).

**Fig. 2:**
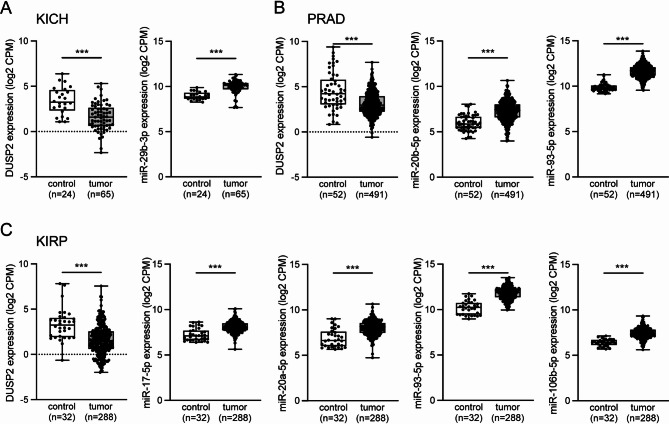
Inverse expression levels of selected microRNA-*DUSP2* pairs between tumour samples and healthy controls derived from the TCGA database. (**A**) In kidney chromophobe (KICH) *DUSP2* showed significantly lower expression compared to healthy controls while expression levels of miR-29b-3p exhibited an inverse pattern. (**B**) A similar observation was made in prostate cancer (PRAD) where *DUSP2* expression levels were significantly lower in tumour tissue compared to healthy controls while an opposite effect was found for miR-20b-5p and miR-93-5p. (**C**) Also in kidney renal papillary cell carcinoma (KIRP) *DUSP2* expression levels were significantly lower in tumour tissue compared to control while miR-17-5p, miR-20a-5p, miR-93-5p and miR-106b-5p showed an opposing pattern. Mann–Whitney U-test; *p ≤ 0.05, ***p ≤ 0.001

Furthermore, the analysis of the TCGA data depicted inverse expression levels of microRNA-*DUSP2* pairs between tumours and controls for members of the miR-17-92 and the miR-29 cluster e.g. in kidney or prostate cancer (Fig. 2). Inverse expression was also identified for distinct microRNA-*DUSP2* pairs in other cancer types like bladder or breast cancer (Fig S2). Together with the significant negative correlations between *DUSP2* mRNA and abundance of phosphorylated MAPK proteins determined by RPPA observed in several tumour types (Fig. 1C), these data supported a potential microRNA-mediated *DUSP2* regulation in cancer.

### Selected microRNAs interact with the *DUSP2* 3’UTR

Next, an extensive literature search was performed in PubMed for the 27 significant negatively correlated microRNA-*DUSP2* pairs to further explore their potential involvement in MAPK hyperactivation in the related malignancy (Tab. S2). Based on this combined approach of in silico target prediction, pan-cancer correlation analysis and literature search, we selected 13 microRNA-*DUSP2* pairs for further functional validation (Table 2). Luciferase reporter gene assays including site-directed mutagenesis were performed to validate the predicted interactions with the *DUSP2* 3’UTR.

**Table 2:** Candidate MicroRNAs selected for functional validation on the basis of in silico target prediction, pan-cancer correlation analysis and literature search

microRNA	Function/relevance in cancer	References
**miR-17-92 cluster**	- **Members**: miR-17, miR-18a, miR-19a, miR-20a, miR-19b-1, miR-92a-1, known as oncomiR-1 - **Expression**: often overexpressed in hematopoietic / solid cancers, activated by c-myc, MAPK signalling, inhibited by p53 - **Function**: associated with adverse clinical outcome, suppression of multiple tumour suppressor genes, impact on cell cycle, proliferation, apoptosis, angiogenesis	[27, 41, 47, 49, 72]
hsa-miR-17-5p	- **Expression**: upregulated in e.g. lung, breast, stomach, prostate, colon, pancreatic cancer, **renal cell carcinoma**, **brain tumours**, **mesothelioma** - **Function**: highly context depended, mostly considered as oncogene, involved in proliferation and decoupling of negative regulators of MAPK signalling pathway	[61, 62, 73, 74, 83, 110]
hsa-miR-20a-5p	- **Expression**: upregulated in e.g. breast, cervix cancer, liver cancer, **renal cell carcinoma**, **brain tumours**,** mesothelioma** while downregulated e.g. in endometrial cancer and AML - **Function**: context dependent oncogene or tumour suppressor function which affects PI3K-Akt, MAPK and TGF-β signalling, contributes to chemoresistance - DUSP2 is a proven target of miR-20a-5p in endometriosis	[21, 62, 65, 73, 82]
**miR-106a-363 cluster**	- **Members**: miR-106a, miR-18b, miR-19b-2, miR-20b, miR-92a-2, miR-363 - **Expression**: dysregulated in various malignancies - **Function**: involved in angiogenesis, apoptosis, cell growths	[41]
hsa-miR-20b-5p	- **Expression**: upregulated in e.g. **glioma**, **lymphoma**, **uveal melanomas**, **prostate cancer**, sarcoma - **Function**: mostly considered as oncogene involved in proliferation and migration via inhibition of PTEN, inhibition of MAPK signalling and associated with adverse clinical outcome of patients	[41, 64–68, 75, 76]
**miR-106b-25-cluster**	- **Members**: miR-106b, miR-93, miR-25 - **Expression**: upregulated in different cancer types - **Function**: involved in apoptosis, cell cycle progression, proliferation, differentiation, associated with positive regulation of p38 signalling	[41, 50, 85–87]
hsa-miR-93-5p	- **Expression**: oncogene in **glioma**, **sarcoma**,** leukaemia**, cervical, bladder, endometrial, breast, **kidney**,** prostate** cancer, conflicting results in colon cancer - **Function**: promotion of growth, invasion and cancer progression via a MAPK feedback loop - activation of JNK pathway in macrophages by targeting DUSP2	[20, 65, 75, 78, 79, 85, 111, 112]
hsa-miR-106b-5p	- **Expression**: aberrant expression is linked to **glioma**, **sarcoma**, **kidney**, breast, prostate, lung, gastric, colorectal cancer, hepatocellular, oesophageal squamous cell carcinoma, **leukaemia** - **Function**: regulation of target genes involved in tumorigenesis, proliferation, invasion, migration, metastases, associated with adverse clinical outcome and chemoresistance in different cancers	[50, 69–71, 77, 86]
**miR-29-cluster**	- **Members**: miR-29a, miR-29b, miR-29c - **Expression**: dysregulated in various types of cancer - **Function**: often suggested to act as tumour suppressor but in specific cancer contexts also oncogenic functions including regulation of epigenetics, proteostasis, metabolism, proliferation, apoptosis, metastasis, fibrosis, angiogenesis immunomodulation and associated with adverse clinical outcome	[89–92]
hsa-miR-29b-3p	- **Expression**: downregulated in many cancer types including cholangiocarcinoma, glioma, osteosarcoma - **Function**: beside a tumour suppressor by inhibiting tumour cell proliferation, invasion, angiogenesis, chemoresistance promoted tumour progression and drug resistance was observed under specific conditions - supports osteoblast differentiation in mice by binding to murine 3´UTR of osteoblast differentiation inhibitors including DUSP2	[22, 89, 90, 113]
hsa-miR-29c-3p	- **Expression**: upregulation in e.g. AML associated with higher risk of relapse - **Function**: mainly tumour suppressor function by inhibiting gastric cancer cell metastasis, decreasing pancreatic cancer cell invasion and metastasis, contributing to negative regulation of MAPK/JNK, CPEB4/MEK/ERK and FBXO31/p38 axis	[114–117]
**C19MC cluster**	- **Expression**: overexpressed in embryonic development and in type A and type AB **thymomas** - **Function**: unknown in type A and type AB thymoma	[97]
hsa-miR-520a-3p	- **Expression**: overexpressed in type A and type AB **thymomas** - **Function**: inhibition of proliferation, apoptosis and metastasis in nasopharyngeal carcinoma and in ling cancer by targeting MAP3K2	[97, 99, 100]
hsa-miR-520c-3p	- **Expression**: overexpressed in type A and type AB **thymomas** - **Function**: modulation of drug sensitivity	[97, 101]
hsa-miR-122-5p	- **Expression**: dysregulated e.g. in **liver cancer** - **Function: i**nhibition of cell migration, invasion via regulation of MAPK signaling	[106, 107]
hsa-miR-142-5p	- **Expression**: aberrant expression in e.g. solid cancers as breast, ovarian, colorectal, lung cancer, also in leukaemia - **Function**: mostly considered as oncogene with impact on apoptosis, proliferation, invasion, migration, leading to MAPK-signalling inactivation	[38, 118, 119]
hsa-miR-340-5p	- **Expression**: dysregulated e.g. in gastric, breast, colorectal, ovarian cancers - **Function**: context dependent tumour suppressor or oncogenic function involved in proliferation, apoptosis, metastasis, as well as associated with diagnosis, treatment, chemoresistance, prognosis - central role in MAPK regulation including p38 activation	[103–105]
hsa-miR-373-3p	- **Expression**: dysregulated in various cancer types e.g. testicular germ cell tumours, breast, liver cancer - **Function**: context dependent tumour suppressor or oncogenic function, implicated in the regulation of proliferation, apoptosis, senescence, migration, invasion, leads to suppression of MAPK pathway signalling	[120, 121]

Cancer type written in bold: Significant negative correlations of microRNA and DUSP2 expression were found in this study

**Fig. 3:**
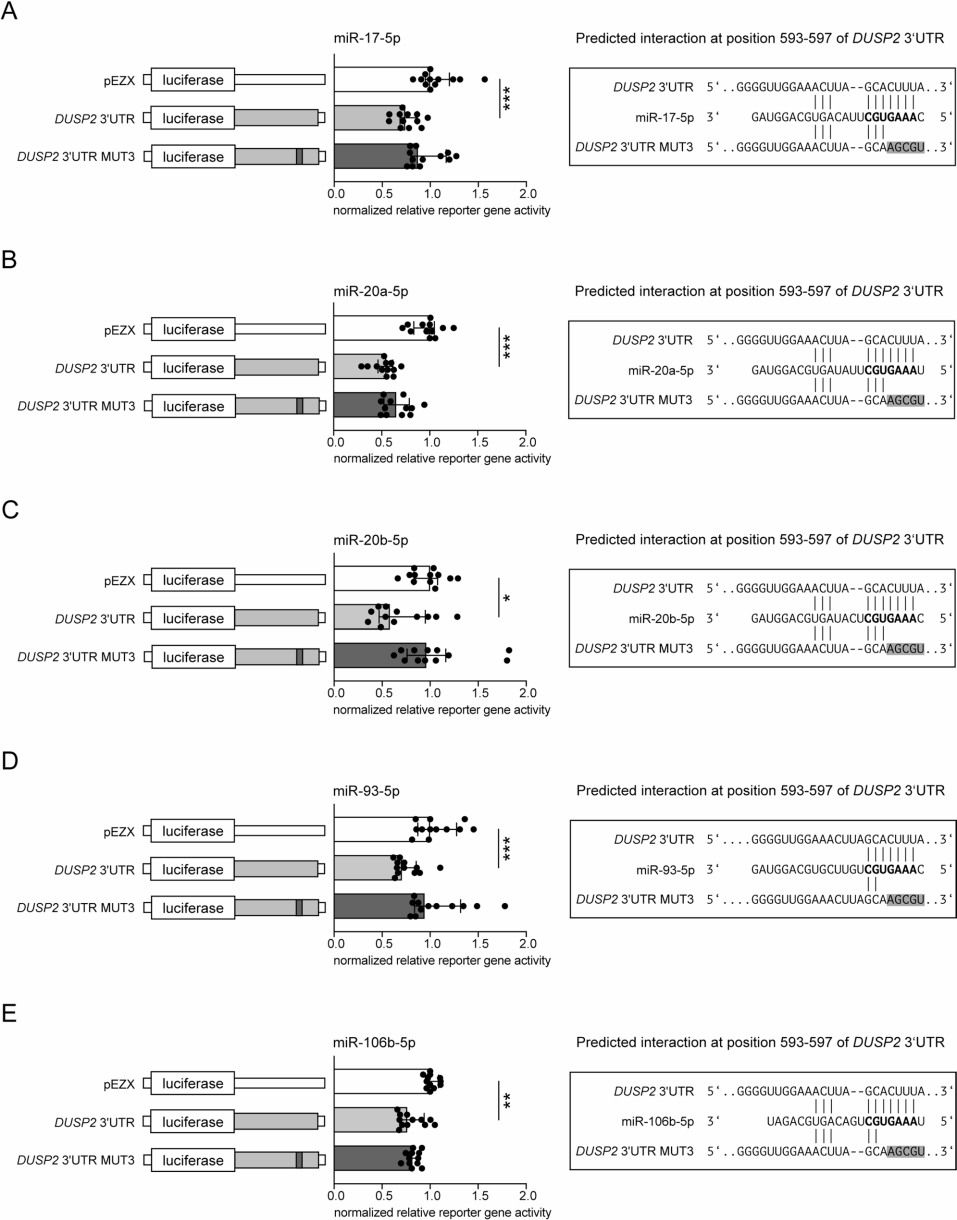
Reporter gene assays indicate direct interactions of miR-17-92 cluster members and members of its paralogues miR-106a-363 and miR-106b-25 with *DUSP2* 3’UTR. Vectors containing the *DUSP2* 3’UTR were co-transfected with 10nM of pre-miR-17-5p, pre-miR-20a-5p, pre-miR-20b-5p, pre-miR-93-5p, pre-miR-106b-5p. Reporter gene activities were measured 48 h after transfection. Transfection with pre-miR-17-5p or pre-miR-20a-5p resulted in suppression of relative reporter gene activity by (**A**) 26% or (**B**) 45%, respectively, through binding to the wild type *DUSP2* 3’UTR. The introduction of mutations (MUT3) into the binding regions neutralized these inhibitory effects (**A**, **B**). **C**) Transfection with pre-miR-20b-5p led to a 42% decrease in normalized reporter gene signal which was abolished by mutations (MUT3) in the binding site. **D**, **E**) Reporter gene assay confirmed the interaction of pre- miR-93-5p and pre- miR-106b-5p with the *DUSP2* 3’UTR by reducing the relative reporter gene activity by 29% and 24%, respectively. While mutations in the predicted binding site completely reversed this effect for pre-miR-93-5p (D) only a partial reversal was observed for pre-miR-106b-5p (E). All activities (*n* = 12) (median ± interquartile range) were shown relative to empty control vector identically transfected and normalized as 3’UTR target sequence vectors. Mann–Whitney U-test; *p ≤ 0.05, **p ≤ 0.01, ***p ≤ 0.001

Reporter gene assays confirmed the interaction of the miR-17-92 cluster members miR-17-5p and miR-20a-5p with the *DUSP2* 3’UTR at positions 593–597 bp by suppressing the normalized reporter gene activity by 26% (*p* < 0.001) for miR-17-5p and by 45% (*p* < 0.001) for miR-20a-5p. This effect was abolished by introducing mutations in the binding sites (Fig. 3A and B).

While the interaction of the miR-106a-363 cluster member miR-106a-5p with the *DUSP2* 3’UTR was previously shown by Qin et al., we identified the interaction of miR-20b-5p with the *DUSP2* 3’UTR, resulting in a 42% decrease in normalized reporter gene signal (*p* = 0.014), which was abolished by mutations at positions 593–597 bp (Fig. 3C) [18].

For two members of the miR-106b-25 cluster, we confirmed the previously described interaction of miR-93-5p with *DUSP2* 3’UTR (29% decrease in normalized reporter gene activity (*p* < 0.001)) and identified the interaction of miR-106b-5p with the *DUSP2* 3’UTR leading to a 24% reduction in normalized reporter signal (*p* = 0.001) [20]. By side directed mutagenesis of position 593–597 bp the binding region was confirmed (Fig. 3D, E).

**Fig. 4:**
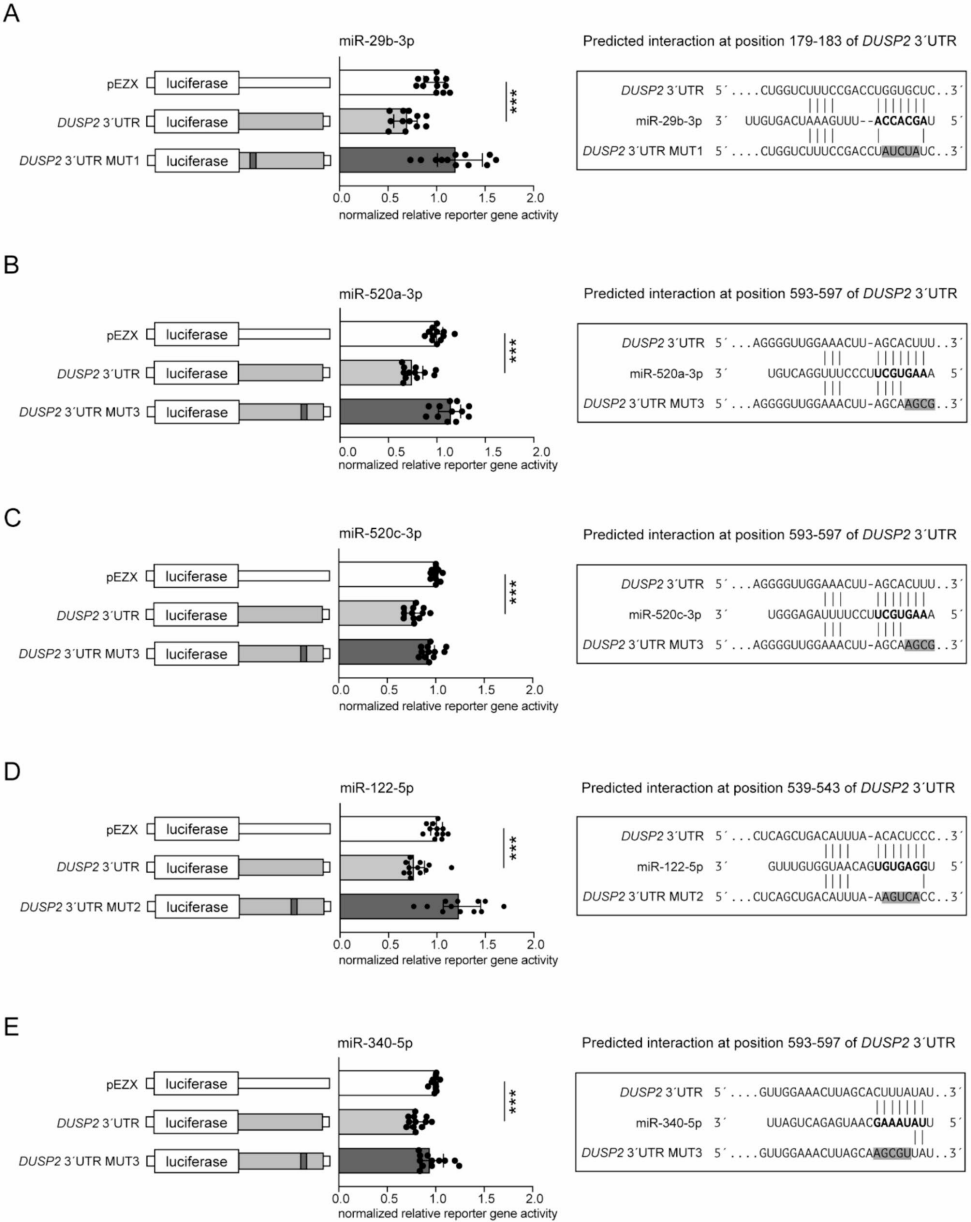
Reporter gene assays determined regulatory effects of miR-29b-3p cluster, members of the C19MC cluster as well as miR-122-5p and miR-340-5p on *DUSP2*. **A**) Reporter gene assay confirmed the interaction of pre-miR-29b-3p (10 nM) with the *DUSP2* 3’UTR by reducing the relative reporter gene activity by 30%. The effects were abrogated by introducing of mutations (MUT1) in the binding site. **B**, **C**) Transfection with pre-miR-520a-3p or pre-miR-520c-3p caused reductions in relative reporter gene activity by 26% or 23%, respectively, through binding to wild type *DUSP2* 3’UTR. The introduction of mutations (MUT3) into the predicted binding region of the *DUSP2* 3’UTR abolished the inhibitory effects of both microRNAs. **D**) Transfection with pre-miR-122-5p also led to reduction of relative reporter gene activity by 24%. The effects were abrogated by introducing mutations (MUT2) in the binding site. **E**) Pre-miR-340-5p transfection resulted in a significant reduction of the relative reporter gene activity by 21%, which was reversed by introducing mutations (MUT3) into the binding region. All activities (*n* = 12) (median ± interquartile range) were determined 48 h after transfection and were shown relative to empty control vector identically transfected and normalized as 3’UTR target sequence vectors. Mann–Whitney U-test; ***p ≤ 0.001

From the miR-29 cluster, miR-29b-3p was confirmed to interact with the *DUSP2* 3’UTR (30% reduction of normalized reporter gene signal (*p* < 0.001), reversible by mutations at positions 179–183 bp) while no interaction was found for miR-29c-3p (Fig. 4A, Fig. S1A).

For the members of the C19MC cluster, miR-520a-3p and miR-520c-3p, reporter gene assay analysis revealed interactions with the *DUSP2* 3’UTR, resulting in a 26% (*p* < 0.001) and 23% (*p* < 0.001) decrease in normalized reporter gene signal, respectively (Fig. 4B, C). Mutation of the predicted binding site at positions 593–597 bp of the *DUSP2* 3’UTR reversed the inhibitory effects of both microRNAs.

The predicted interactions of miR-122-5p (24% (*p* < 0.001) decrease in normalized reporter gene signal) and miR-340-5p with the *DUSP2* 3’UTR were also confirmed at position 539–543 bp and 539–543 bp, respectively (Fig. 4D, E). However, for miR-142-5p and miR-373-3p, the in silico predicted binding to the *DUSP2* 3’UTR could not be confirmed (Fig S1B, C).

### Functional validation of microRNA– *DUSP2* interactions in a DLBCL cell line

**Fig. 5:**
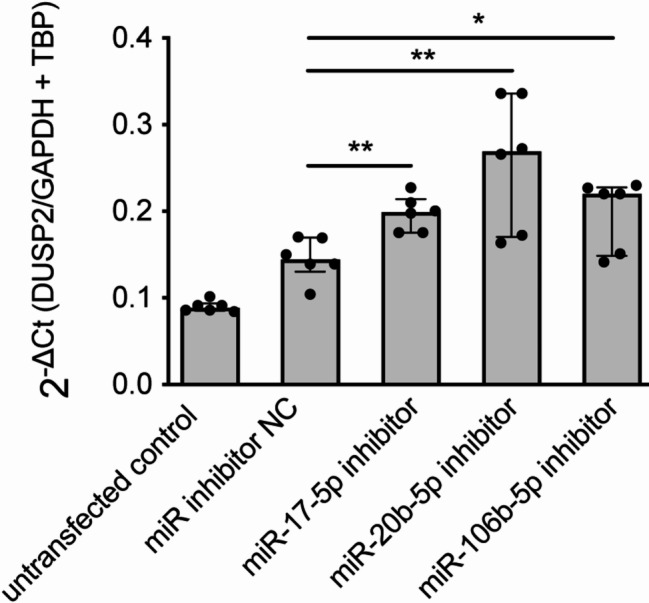
MiR-17-5p, miR-20b-5p and miR-106b-5p regulate *DUSP2* mRNA expression in a lymphoma cell line model

In the next step, the influence of selected microRNAs (miR-17-5p, miR-20b-5p, miR-106b-5p) on *DUSP2* mRNA level was validated in WSU-DLCL2 cells a cell line derived from a diffuse large B-cell lymphoma (DLBCL). A DLBCL cell line was chosen because *DUSP2* was highly expressed in DLBCL (TCGA data, Fig. 1B), though lower than in healthy controls (Fig. S3) and is considered as molecular hallmark for DLBCL subtyping, thereby suggesting a critical role for DUSP2 in DLBCL [42]. The transfection of 100 nM miR-17-5p inhibitor in WSU-DLCL2 cells resulted in a 1.4-fold (*p* = 0.002) increase in *DUSP2* mRNA expression compared to negative control, while transfection with miR-20b-5p inhibitor led to 1.9-fold (*p* = 0.009) and transfection with miR-106b-5p inhibitor to 1.5-fold (*p* = 0.035) increase of *DUSP2* mRNA levels (Fig. 5). These results confirm the microRNA-*DUSP2* interaction in different cell types and a significant impact of various microRNAs on *DUSP2* mRNA levels.

Transfection experiments with 100 nM microRNA inhibitors or control led to a significant increase of *DUSP2* mRNA in WSU-DLCL2 cells. Mann–Whitney U-test; *p ≤ 0.05, **p ≤ 0.01.

## Discussion

Although kinase inhibitors are essential drug components in the treatment of a number of malignancies, their limited long-lasting clinical effect has prompted discussions about further therapeutic strategies [3, 43]. This includes the concept of tumour suppressor phosphatase regulation, which is often compromised by nongenetic cancer-specific mechanisms [3]. DUSP2 is one of these essential negative regulators of the MAPK pathway which ensures a tight and efficient control of MAPKs under physiological conditions, and these genes are strictly regulated themselves e.g. by RNA-binding proteins or microRNAs [44]. In oncogenic context, the balance between signal induction and inactivation is frequently disrupted, which often leads to hyperactivation as signalling observed for the MAPK pathway [1]. Methylation or hypoxia-mediated regulation of DUSP2 has been widely discussed factors contributing to the impairment of DUSP2 function, but it has been found that the negative feedback disruption is not entirely attributable to these mechanisms [7, 8, 21, 45]. The aim of the present study was therefore to investigate potential microRNA-mediated DUSP2 regulation in cancer, as DUSP2 has recently being identified as microRNA target known to be widely dysregulated in cancer and to contribute to tumorigeneses [16–25]. By combining in silico target prediction, pan-cancer correlation analysis and literature search, we identified six novel and confirmed four previously described interactions between microRNAs and *DUSP2*. The data support the hypothesis that alterations of microRNA expression in cancer may contribute to DUSP2 dysregulation.

In the present study, significant negative correlations were found between members of microRNA clusters miR-17-92 (miR-17-5p, miR-20a-5p), miR-106a-363 (miR-20b-5p, miR-106a-5p), miR-106b-25 (miR-93-5p, miR-106b-5p) which are regarded as oncogenic in hematopoietic malignancies and solid tumours and *DUSP2* expression in a number of malignancies derived from the TCGA database [41, 46–51]. According to the available data, those types of cancer partly exhibited MAP-kinase pathway hyperactivation even in the absence of genetic driver alterations, suggesting alternative mechanisms of activation, including microRNA alterations, epigenetic changes or alternative splicing [52–60]. Indeed, for the six microRNAs investigated in our study (miR-17-5p, miR-20a-5p, miR-20b-5p, miR-106a-5p, miR-93-5p and miR-106b-5p) an overexpression and oncogenic potential were described (Tab. S1). The reports included associations with poor prognosis and clinical outcome such as advanced tumour grade, decreased overall survival and increased risk for relapses or chemoresistance (Table 2) [50, 61–71]. Furthermore, the overexpression of the microRNAs was associated with the downregulation of tumour suppressor genes as well as increased tumour proliferation and invasion [50, 72–81].

In this study, we confirmed the previously described miR-20a-5p–*DUSP2* interaction which was observed to increase ERK activity and contribute to chemoresistance [21, 82]. Furthermore, we confirmed the interaction of miR-17-5p with *DUSP2* 3’UTR which was previously reported to be highly expressed and associated with poor prognosis in gliomas and described as efficiently de-coupling other negative regulators of the MAPK pathway [61, 83]. Thus, the miR-17-5p-mediated regulation of *DUSP2* might provide a further component in the negative regulatory network of MAPK signalling. Additionally, miR-20b-5p, a member of the miR-106a-363 cluster was identified in our study to interact with *DUSP2*. MiR-20b-5p is reportedly associated with cancer progression by inhibiting another tumour suppressor phosphatase, namely PTEN [76, 84]. Moreover, we demonstrated that inhibition of miR-20b-5p in the DLBCL cell model leads to a significant increase in *DUSP2* mRNA expression levels. MiR-106a-5p, another member of the miR-106a-363 cluster involved in DUSP regulation was previously shown to be associated with 5-fluorouracil resistance [18].

With respect to the miR-106b-25 cluster, we confirmed the interaction of miR-93-5p with the *DUSP2* 3’UTR [20] and additionally identified miR-106b-5p targeting *DUSP2*. For both microRNAs associations with positive regulation of p38 signalling have been reported in various cancer types [85–87]. Although a direct binding of miR-93-5p to MAP3K2 might contribute to p38 regulation, the underlying mechanism is not fully understood so far [85, 88]. Thus, the direct regulation of *DUSP2* by miR-106b-5p and miR-93-5p might provide another component in the regulatory network of MAPKs in respective cancer types and requires to be further investigated. Additionally, for miR-106b-5p we were able to show that inhibition of this microRNA in the DLBCL cell model leads to significant increase in *DUSP2* mRNA levels.

The miR-29 cluster is often suggested to act as tumour suppressor, but in specific cancer contexts, some cluster members exhibited also oncogenic functions [89]. In our pan-cancer correlation analysis we found significant negative correlations of miR-29 cluster members with *DUSP2* expression in various cancer types such as adrenocortical carcinoma, bladder, colorectal and kidney cancer, mesothelioma and uveal melanoma, but most prominent in thymoma. Here, the *DUSP2* expression was also significantly negative correlated to phosphorylation levels of ERK2 and p38. Oncogenic overexpression of miR-29 cluster members have been associated with adverse clinical outcome as well as tumour progression and drug resistance via direct suppression of the phosphatase PTEN (Table 2) [90–96]. Furthermore, we found several members of the large C19MC microRNA cluster to be significantly negatively correlated to *DUSP2* expression, exclusively in thymomas [97, 98]. Under physiological conditions this microRNA cluster is predominantly expressed in embryonic development and silent in adult tissue except for placenta, but it was identified as a genomic hallmark of type A and type AB thymomas with unknown function (Table 2) [97, 99–101]. Since a recent study described MAPK hyperactivation as a new hallmark of type A and AB thymomas, the here identified direct interaction of two cluster members (miR-520a-3p, miR-520c-3p) with *DUSP2* as well as of miR-29 cluster members might contribute to MAPK activation via repression of negative regulators [102].

MiR-340-5p, a microRNA with context-dependent tumour suppressor or oncogenic functions, was significantly negatively correlated to *DUSP2* expression in various cancer types like in thymoma, brain tumours, colorectal and bladder cancer as well as in lymphoma [103]. Moreover, several studies indicated a central role of miR-340-5p in MAPK regulation including p38 activation (Table 2) [103–105]. Besides the miR-340-5p-mediated regulation of MAP3K2 or direct interaction with p38, the identified interaction of miR-340-5p with *DUSP2* 3’UTR might be another mechanism contributing to the p38 activation which needs further analysis in future studies [104, 105].

Likewise, the newly identified interaction between mir-122-5p and *DUSP2* should be the subject of future studies since a significant negative correlation was found in hepatocellular carcinoma while miR-122-5p is primarily regarded as a liver-specific tumour suppressor i.a. by downregulation of DUSP4 (Table 2) [106, 107].

Although we perform a rigorous preselection of potential microRNA-*DUSP2* pairs for validation experiments which based on the combined approach and confirmed the majority of the putative interactions, we found no interactions for the predicted binding of miR-29c-3p, miR-142-5p, and miR-373-3p to *DUSP2* 3’UTR. This observation was not unexpected since target prediction algorithms have a known false positive rate of 20–50% [108]. In fact, these results emphasize that a combined approach provides more robust indication for real microRNA– target gene interactions compared to sole target prediction, as seen for miR-142-5p and miR-373-3p. However, it must be taken into account that the analysed number of available microRNA and *DUSP2* expression as well as MAPK phosphorylation data varied markedly between the TCGA cancer types and normal control data were not available for all cancer types. Additionally, DUSP2 is one negative regulator of the MAPKs, which might act in concert with other factors in regulating the pathway. Moreover, the identified microRNA–*DUSP2* interactions were verified in an in vitro setup and exemplarily studied in a DLBCL cell model due to the potential crucial role of DUSP2 in DLBCL. Hence, further substantiation of these findings is required by functional studies analysing the direct effect of microRNA-mediated *DUSP2* regulation on MAPK activity in the respective cancer type as microRNA–target gene interactions are known to be dynamic and modularized mechanisms which may differ significantly between specific intracellular and cancer contexts [109].

In summary, through in silico research and in-vitro experiments, we could identify and confirmed novel and earlier described oncogenic microRNA–*DUSP2* interactions putatively leading to dysregulation of MAP kinase pathways in a number of malignancies. The findings can be used as solid foundation to further investigate disruption of negative feedback mechanisms in various types of cancer. These results may also serve as basis for more comprehensive investigations aiming at fully elucidating the regulation of *DUSP2* by cancer-associated microRNAs and to further evaluate the tumour suppressive potential of DUSP2 reactivation.

## Electronic supplementary material

Below is the link to the electronic supplementary material.

Supplementary Material 1

Supplementary Material 2

Supplementary Material 3

## Data Availability

The microRNA and DUSP2 expression data as well as the MAPK and DUSP2 protein data from 32 cancer types which were analysed within this study were extracted from the CancerMIRNome database, The Cancer Genome Atlas Program (TCGA) (www.cancer.gov/tcga) and cbioportal. Li R, Qu H, Wang S, Chater JM, Wang X, Cui Y, Yu L, Zhou R, Jia Q, Traband R et al.: CancerMIRNome: an interactive analysis and visualization database for miRNome profiles of human cancer. Nucleic Acids Res 2022, 50(D1):D1139-d1146. Cerami E, Gao J, Dogrusoz U, Gross BE, Sumer SO, Aksoy BA, Jacobsen A, Byrne CJ, Heuer ML, Larsson E et al.: The cBio cancer genomics portal: an open platform for exploring multidimensional cancer genomics data. Cancer Discov 2012, 2(5):401-404. Gao J, Aksoy BA, Dogrusoz U, Dresdner G, Gross B, Sumer SO, Sun Y, Jacobsen A, Sinha R, Larsson E et al.: Integrative analysis of complex cancer genomics and clinical profiles using the cBioPortal. Sci Signal 2013, 6(269):pl1.
