# Supplementary material for: The MAP kinase negative regulator DUSP2 (dual specificity phosphatase 2) is controlled by oncogenic microRNA cluster miR-17-92, miR-106a-363 and miR-106b-25
Source: BMC Cancer. 2025 Jun 19;25:1020. doi: 10.1186/s12885-025-14434-z (PMC12180267; doi:10.1186/s12885-025-14434-z)
Supplement: Supplementary file 3 — Supplementary Material 3 [file 12885_2025_14434_MOESM3_ESM.docx]

Supplementary Material

**The MAP kinase negative regulator DUSP2 (dual specificity phosphatase 2) is controlled by oncogenic microRNA cluster miR-17-92, miR-106a-363 and miR-106b-25**

**Victoria Tenhaken^1^, Ole-Morten Seternes^2^, Ingolf Cascorbi^1^, Henrike Bruckmueller^1,2^**

*** Correspondence:** Henrike Bruckmueller: h.bruckmueller@pharmakologie.uni-kiel.de

**Table S1**

Excel table

**Combined approach of *in silico* target prediction and pan-cancer correlation analysis exhibiting all significant correlations for all 83 predicted candidate microRNAs**

**Table S2**

Excel table

**Literature review for the 27 microRNA-DUSP2 pairs showing significant negative correlations in at least on cancer type with focus on microRNA role and MAPK hyperactivity in the respective cancer type as well as similarity of seed sequences to microRNAs already described to interact with DUSP2**

**Fig. S1**

**Reporter gene assays exhibited no interaction between miR-29c-3p, miR-142-5p and miR-373-3p and DUSP2 3´UTR.** A) Reporter gene assays resulted in no significant suppression of relative reporter gene activity when transfected with pre-miR-29c-3p (A), miR-142-5p (B) or miR-373-3p (C)**.** All activities (n = 12) (median ± interquartile range) were determined 48h after transfection and were shown relative to empty control vector identically transfected and normalized as 3′-UTR target sequence vectors. Mann–Whitney U-test; ^ns^p > 0.05.

**Fig. S2**

**Comparison of expression levels of microRNA-*DUSP2* pairs between tumour samples and healthy controls derived from the TCGA database.** A) In bladder urothelial carcinoma (BLCA) the expression of *DUSP2* was significantly lower compared to healthy controls, while the expression levels of miR-455-3p showed an inverse picture. B) In breast invasive carcinoma (BRCA) a similar trend was seen in DUSP2 expression levels, while the expression of miR-149-5p was significantly higher in tumor samples. C) While the difference of DUSP2 expression between head and neck squamous cell carcinoma (HNSC) and controls was not significant, miR-455-3p and miR-1910-5p showed significantly higher expression in tumor samples. In liver hepatocellular carcinoma (LIHC) (D) and thyroid carcinoma (THCA) (F) DUSP2 expression levels were significantly lower in tumor samples than in controls, while no significant differences were found for stomach adenocarcinoma (STAD) (E) or uterine corpus endometrial carcinoma (UCEC) (G). No inverse correlation of potential microRNAs was found for the latter cancer types (Mann–Whitney U-test; *p ≤ 0.05, **p ≤ 0.01, ***p ≤ 0.001).

**Fig. S3**

**
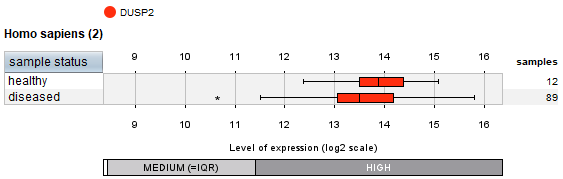
**

**Comparison of DUSP2 mRNA expression in DLBCL samples and healthy controls**

The mRNA expression levels of DUSP2 in DLBCl samples and peripheral blood B-cells obtained from healthy volunteers were extracted from the Genevestigator database. Expression values are given in log2 scale arbitrary units according to Genevestigator [1]. The presented data are part of the dataset from the study of Dybkær et al.2015 [2]

1. Hruz T, Laule O, Szabo G, et al. Genevestigator v3: a reference expression database for the meta-analysis of transcriptomes. Adv Bioinformatics. 2008;2008:420747.

2. Dybkær K, Bøgsted M, Falgreen S, et al. Diffuse large B-cell lymphoma classification system that associates normal B-cell subset phenotypes with prognosis. J Clin Oncol. 2015 Apr 20;33(12):1379-88.
